# Supplementary figures and images for: PSMA-Targeted Radiolabeled Peptide for Imaging and Therapy in Prostate Cancer: Preclinical Evaluation of Biodistribution and Therapeutic Efficacy
Source: Int J Mol Sci. 2025 Aug 5;26(15):7580. doi: 10.3390/ijms26157580 (PMC12346970; doi:10.3390/ijms26157580)

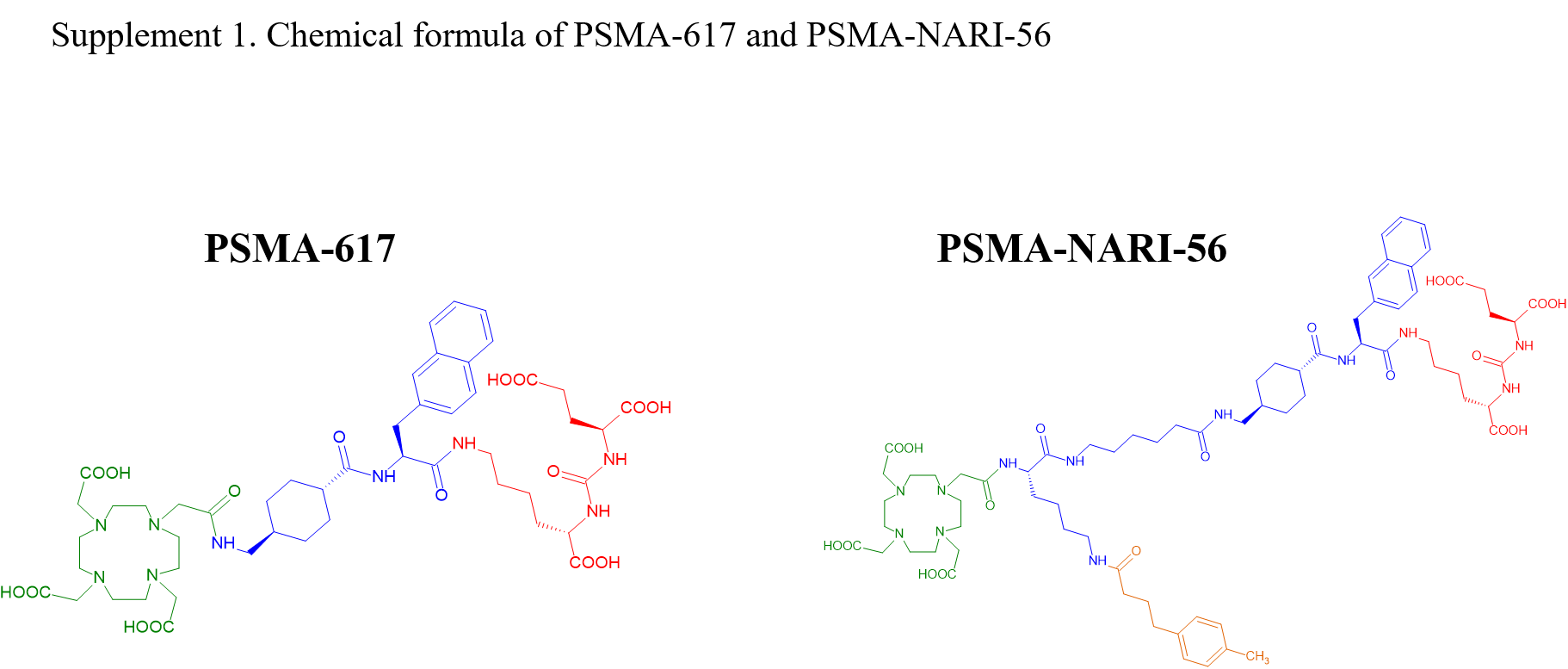

Supplement: Supplementary file 1 [file ijms-26-07580-s001.zip › Supplement 1.png]

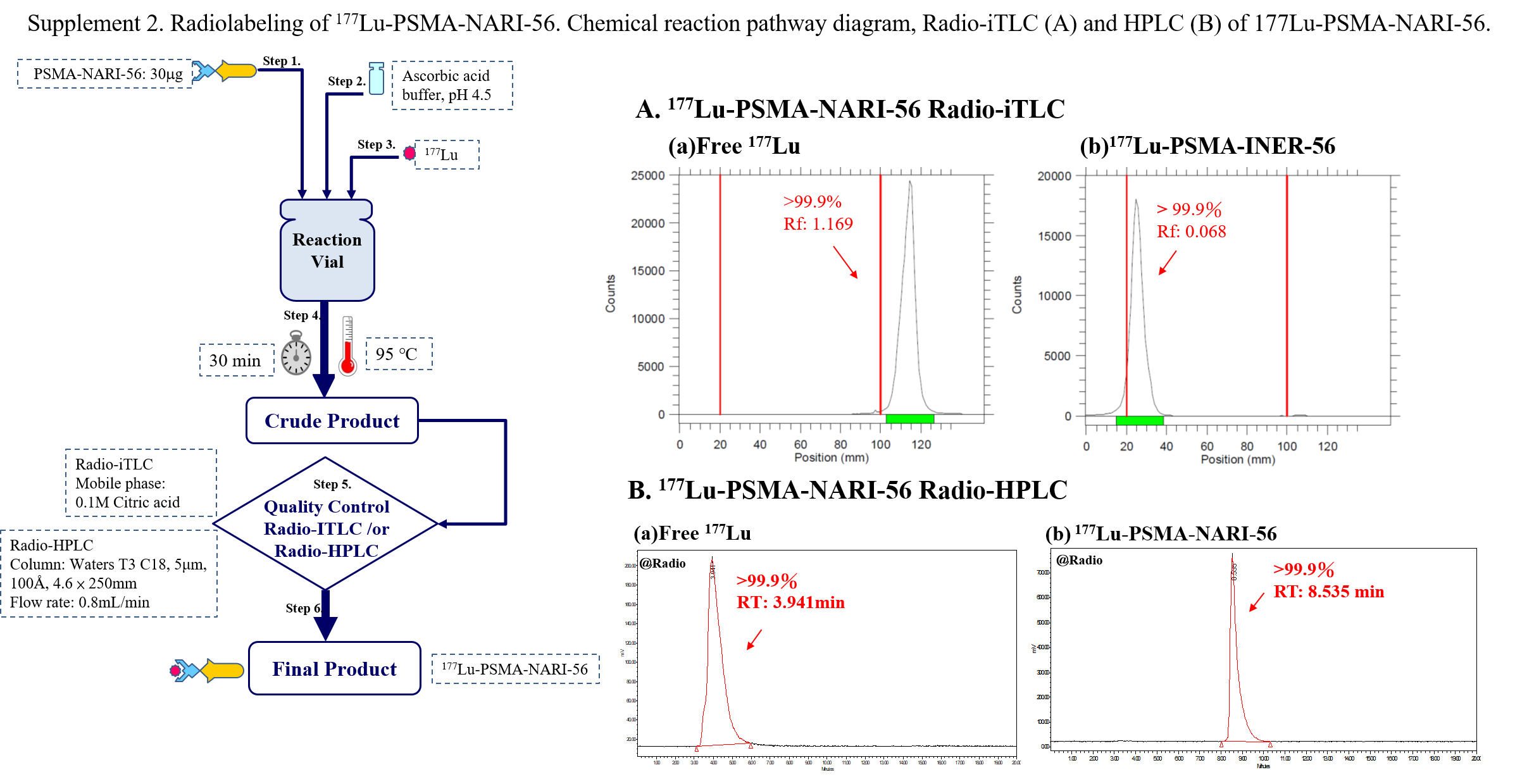

Supplement: Supplementary file 1 [file ijms-26-07580-s001.zip › Supplement 2.png]
